# Research Performance Measures and the Moderating Role of Faculty Characteristics in Epidemiology

**DOI:** 10.5539/gjhs.v8n5p72

**Published:** 2015-08-31

**Authors:** Maryam Okhovati, Azam Bazrafshan, Morteza Zare, Mina Moradzadeh, Ali Mohammad Mokhtari

**Affiliations:** 1 Physiology Research Center, Institute of Neuropharmacology, Kerman University of Medical Sciences, Kerman, Iran; 2 Neuroscience Research Center, Institute of Neuropharmacology, Kerman University of Medical Sciences, Kerman, Iran; 3 Health Services Management Research Center, Institute for Futures Studies in Health, Kerman University of Medical Sciences, Kerman, Iran; 4 School of Public Health, Shiraz University of Medical Sciences, Shiraz, Iran

## Abstract

Several numeric measures have been proposed to evaluate the individual researchers’ scientific performance. Among these measures, h-index is the most common and well recognized measure of research productivity and impact in scientific communities. However, empirical investigations and recent inspections revealed some shortcomings and limitations of this measure. In order to complement these limitations, several variants have been proposed in which g-index and ar-index were among the most discussed measures. The aim of this study was to examine h-index, g-index and ar-index across Iranian epidemiologists to identify the moderating characteristics as well as the distribution of these measures in the field. Using Web of Science Database, a list of Iranian epidemiologists was searched and total number of articles, total citations, and citations per paper, h-index, scientific age, g-index and ar-index were extracted and calculated for any epidemiologist. Descriptive statistics and multivariate linear regression models were used to examine research performance measures of Iranian epidemiologists. According to our findings, research performance measures found to be statistically associated with scientific age and academic ranking of Iranian Epidemiologists. Gender differences were not relevant to research performance across different measures.

## 1. Introduction

There is a growing recognition of the needs for measuring the quality, performance and impact of individual researchers when making decisions on promotion, research grant allocation and recruiting academic staff. In order to address these needs; previous efforts have been made over the years to propose numeric measures of individual’s research impact as well as productivity. Total number of publications, total citation count and citations per publication are among the most common used measures for a long time (Bornmann & Daniel, 2009).

Hirsch (2005) proposed h-index, a widely accepted and used measure of research performance and an alternative to previous measures (Bornmann, Mutz, Hug, & Daniel, 2011). “A scientist has index *h* if *h* of his or her *N_p_* papers have at least *h* citations each and the other (*N_p_*– *h*) papers have ≤*h* citations each” (Hirsch, 2005). In other words, when a scientist’s h index is 7, it means he/she has 7 papers which have been cited at least 7 times. Today, h-indexis a widely accepted and used measure of research performance.

Various studies have reported h-index for scientists in different fields (Malesios & Psarakis, 2014; Rad, Brinjikji, Cloft, & Kallmes, 2010; Pashkova et al., 2013; Oppenheim, 2007; Healy et al., 2011; Batista, Campiteli, & Kinouchi, 2006; Lacasse, Hodge, & Bean, 2011; Babineau, Fischer, Volz, & Sanchez,2014; Doja et al., 2014). Several efforts have revealed the significant implication of h-index in measuring individual researchers’scientific productivity and impact (Babineau et al., 2014; Svider et al., 2013; Wilkes et al., 2015; Therattil, Hoppe, Granick, & Lee, 2014). In this regard, some advantages have been attributed to h-index such as its simplicity (Franceschini & Maisano, 2010), objectivity (Costas & Bordons, 2007), robustness against highly cited and low or no cited papers (Waltman & Van Eck, 2012; Bornmann, Mutz, & Daniel, 2008) and suitability for assessment of small paper sets (Glanzel, 2006). Moreover, it is a single number combining quantity and quality (Hirsch, 2007; Mingers, 2009) and is calculated automatically in WOS, Scopus, and Google Scholar (Bar-Ilan, 2008).

A number of disadvantages have also been proposed in previous studies, Bornmann et al. (2011) point out that h-index is field-dependent, influenced by multi-authorshipand self-citations. H-index considers quantity more than anything else (Antonakis & Lalive, 2008) and is useful for comparing the better researchers in a field not average scientists (Jian & Xiaoli, 2013). Furthermore, h-index value never decreases over time as it “is highly dependent upon a scientist’s number of years of active research” (Bornmann et al., 2008).

In order to address these limitations and overcome the h-index shortcomings, several variants have been developed. Ar-index has been suggested by Jin, Liang, Rousseau and Egghe (2007) to complement h-index by taking the actual number of citations into account, and also considering the age of the publications. Jin et al. (2007) proposed the pair of the h and arindices as a meaningful indicator for research evaluation. G-index has been proposed as another measure of scientific performance, Schreiber (2008) believes that the g-index is more suitable than h-index to characterize the total impact of the publications of a scientist and it differentiates better between different citation patterns. If the papers are arranged in the descending order of their number of citations, g-index is the largest number such that the summation of the number of citations is at least g^2^ (Abbas, 2011). De Visscher (2011) argues that h-index ignores consistency of impact while the g-index considers this consistency. Egghe (2012) presenting an example illustrates thatg-index rewards authors having a high number of citations even if they have published a very few publications.

Bornmann et al. (2011) carried out a meta-analysis on studies reporting correlation between h-index and 37 h-index variants. They have concluded that h-index variants make a non-redundant contribution to h-index. Another study has revealed that there are two independent types of h-index variants. The first group consists of those variants that describe the number of papers in the most productive core (h index, m quotient, g index and h(2) index) and the second type of indices (a index, m index, r index, ar index and hw index) represents the impact of papers in the core. It was concluded that the two types of indices can complement each other (Bornmann et al., 2008).

Today, h-index is widely used in different disciplines particularly in biomedical and health care sciences (Jin et al., 2007) and is found better than other measures in predicting future scientific achievement (Hirsch, 2007). Since the h-index and its complementing measures are not suitable to compare researchers in different fields, it is necessary for each field of science to develop its own norms (Doja et al., 2014). H-index and its well documented variants are among the primary tools in measuring the individual’s research performance, thus the present study is to examine the most important h-index variants that have been discussed in the literature including h-index, g- index and ar-index in the field of epidemiology. The findings of this study can represent the impact of Iranian epidemiologists on this field using significant indices and provides a way to track their scientific progress and impact.

## 2. Methods

To find the Iranian epidemiologists papers in Web of Science Database (WOS), *epidemiology* keyword limited to Iran in address field was searched. In this way, a list of publications affiliated by Iranian epidemiologists was achieved. The authors’ names were checked with the Iranian Association of Epidemiologists to include all epidemiologists and exclude irrelevant individuals. Finally, the publications of ninety-one Iranian epidemiologists were searched in WOS by August 2013. If a name had various spelling, all variations were searched and to get sure all the papers belonged to the same person, we checked the ID number of researchers in the database. Then total number of papers and citations per paper were extracted and the h index was calculated for each person. The g and ar indices were manually obtained according to the following formulas:

Ar-index (Bornmann et al., 2008):


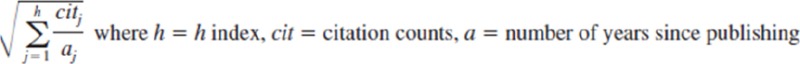


g-index, the highest number g of papers that together received g^2^ or more citations (Thompson, 2009):


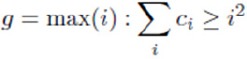


Scientific age was considered as the years after the first publication in WOS Database. Data on demographic information including gender and academic rank of the epidemiologists were obtained using available records of the Iranian Association of Epidemiologists.

Descriptive statistics (Mean, Standard Deviation, Maximum, and Minimum) used to represent the overall impact of Iranian epidemiologists in the field. Correlation tests, and adjusted linear regression model was used to investigate the moderating role of epidemiologists’ characteristics. The significance level was 0.05 for two sided tests.

## 3. Results

A total of 91 epidemiologists (all Iranian epidemiologists who had papers in WOS) including 77 males and 14 females were studied through census. According to their academic ranking groups, 10 instructors, 39 assistant professors, 28 associate professors and 14 full professors were included in the study.

Totally, Iranian epidemiologists published 1860 papers. The mean (median) of papers published was 20.4 (16). The maximum of papers published was 98 and the minimum was 0. The highest scientific age of Iranian epidemiologists and its mean was 37 and 8.4, respectively. The highest h-index was 17 and the mean (median) was 3.9 (4), whereas the highest g-index calculated was 28, with the mean (median) of 6.8 (6). The maximum of ar-index (29.6) was higher than the maximum of h-index and g-index but the mean of g-index was higher than two other measures.

Table 1 shows the statistical values of Min, Median, Max and Mean of the total papers, H index, G index, AR index and scientific age for Iranian epidemiologists.

**Table 1:** The distribution of Iranian Epidemiologists’ scientific measures in Web of Science Database

indicators	Total papers	H index	G index	AR index	Scientific age
***Min***	0	0	0	0	0
***Median***	16	4	6	4.2	7
***Max***	98	17	28	29.6	37
***Mean***	20.4	3.9	6.8	5.5	8.4

**Figure 1:**
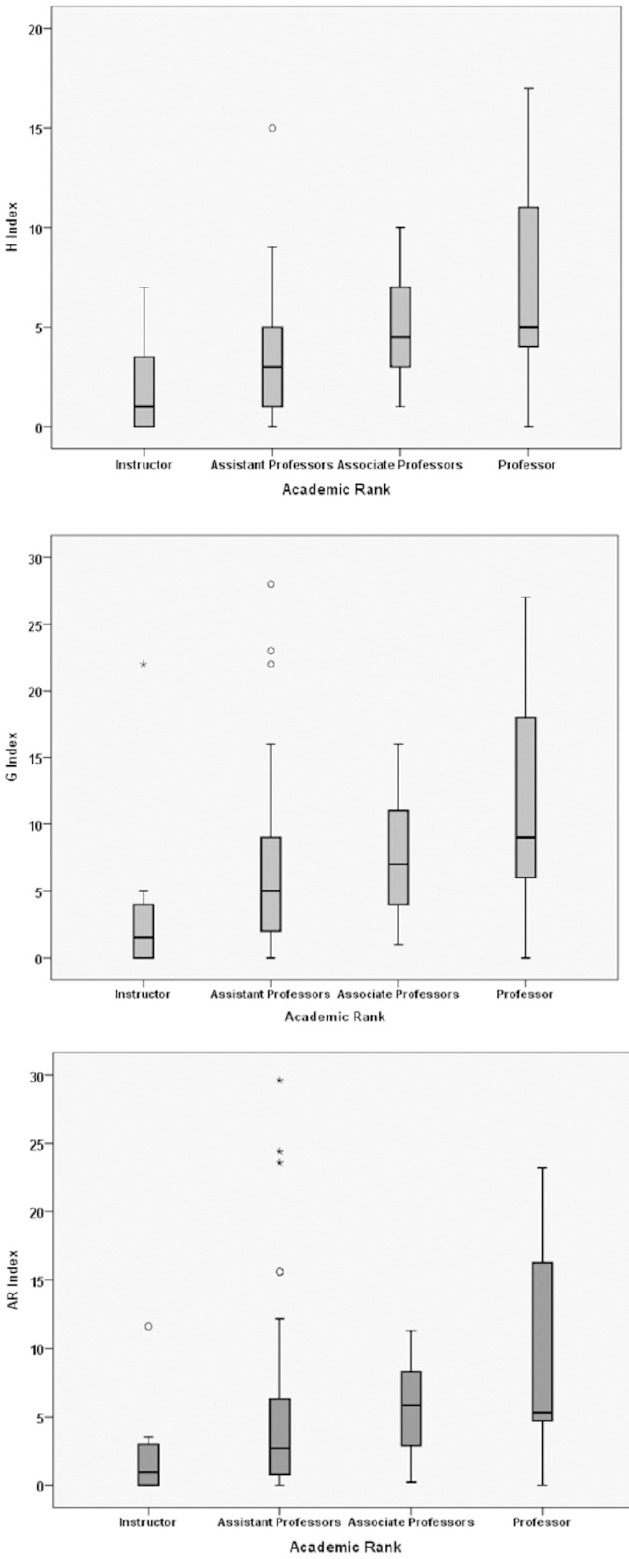
Distribution of Iranian Epidemiologists’ research performance measures according to their academic rankings

Figure 1 illustrates the distribution of h-index, g-index and ar-index according to the Iranian Epidemiologists’ academic ranking. According to this figure, research performance of Iranian epidemiologists developed aligned with improving academic rankings. In this regard, professors received higher scores than associate professors and assistant professors, across h-index and g-index significantly. However, ar-index followed rather a different pattern in which associate professors received higher scores than professors. According to this figure, a significant difference was observed among academic ranks (p=0.003). This difference was significant between instructors and assistant professors (p=0.001), assistant professors and professors (p=0.001). But no significant difference was found between assistant professors and associate professors.

**Figure 2:**
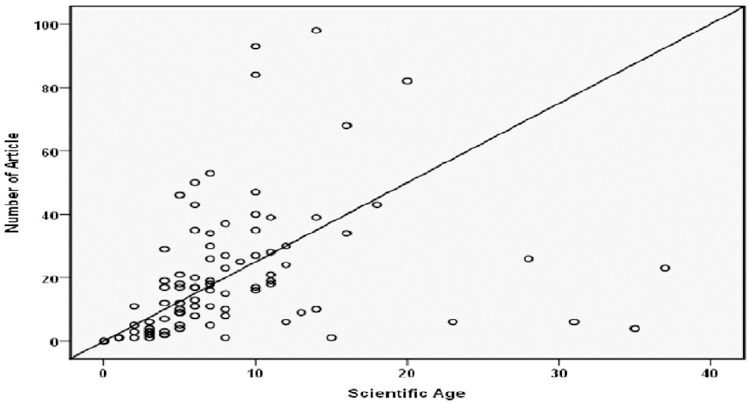
Distribution of Iranian Epidemiologists’ Scientific age according to the number of papers

Our findings also indicated a correlation between scientific age, the number of published papers (r=0.27, p=0.008) and h index (r=0.32, p=0.002) (Figure 2).

Further inspection of epidemiologists’research performance revealed that fixed linear effect of scientific age was statistically significant across h-index (p=0.04), g-index (p=0.06) but not ar-index (p=0.30). This means that scientific age of epidemiologists did not have positive association with their research performance measures. Although men received higher performance measures than women across different measures, their performance scores did not seem to vary significantly. Professors received higher adjusted scores than other academic rank groups across different performance measures (Table 2).

**Table 2:** Prediction of Iranian epidemiologists’ research performance measures according to multivariate linear regression analysis

	H Index		G Index		AR Index	

	β	P value	β	P value	β	P value
**scientific age**	0.1	0.04	0.19	0.06	0.01	0.3
**sex**						
female	Ref.	--	Ref.	--	Ref.	--
male	1.36	0.17	2.35	0.22	0.34	0.27
**Academic ranking**						
instructors	Ref.	--	Ref.	--	Ref.	--
assistant professors	0.99	0.43	1.5	0.43	0.45	0.25
associate professors	1.83	0.17	1.9	0.17	0.37	0.37
professors	4.16	0.007	5.76	0.03	0.95	0.04

## 4. Discussion

The key finding of the present study was positive associations between the Iranian epidemiologists’ scientific age and their h-index, g-index and ar-index. Iranian Epidemiologists seemed to have similar performance according to their gender across different research performance measures. Professors received significantly higher adjusted scores than other academic rank groups across measures.

According to our findings, Iranian epidemiologists published nearly two thousand papers in WOS database by August 2013. The overall examination of epidemiologists’ publications implied that the data distribution was skewed and was not close to the mean. A comparison of Iranian Epidemiologists’ publishing patterns with other research fields indicated that Iranian microbiologists published more papers in journals indexed in Web of Science Database (Ramezani, Nikokar, Bandboni, & Rohi, 2014). Iranian scientists in the field of chemistry were even more active, so that they published the highest number of papers during 1996-2006 compared with other research areas (Aminpour & Kabiri, 2009).

The mean of scientific age indicates that the epidemiologists were relatively young once publishing papers in WOS. The findings showed that the number of papers and citations were increasing annually, so that they would improve their productivity in future.

The top h-index for Iranian epidemiologists was 17. The mean g-index of them was 6.8 versus the mean h-index, 3.9. In contrast with h-index, g-index gives weight to highly cited papers, and it is clear that it is higher than h-index (Bornmann et al., 2008). So the mean g-index of epidemiologists has considered paper cited out of range.

The mean ar-index was 5.5, according to this measure if any epidemiologists were not active as before, this index decreased but it seems as a whole, Iranian epidemiologists are active. We found a positive association between scientific age and the h-index. As the scientific age increased the chance for increasing h-index also improved. It implies that those with more scientific age have more experience in writing high quality papers and then have more chance to be cited. On the other hand, h- index had no significant association with epidemiologists’ gender but had significant association with academic rank.

A comparison of researchers in different research areas indicated that the top h-index for breast cancer researchers was 80. Among the health service researchers in some countries, statisticians had the highest h-index (the median was 17). The highest h-index rank for scientists in the field of energy was 57 (Budzianowski, 2014). Babineau et al. (2014) reported h-index for a number of specialties; the mean h-index was 12.5for emergency medicine in WOS, and 16 through Google Scholar, 9 for anaesthesia, 22 for urology, 12 for CT anaesthesia, 12.5 for radiology and 15.6 for ENT. Batista et al. (2006) examined the h-index for Brazilian scientists; they reported that the top h-index for Brazilian physicians and chemists was 37 and 29, respectively. The highest h-index for mathematicians was 14. H-indices 0 and 1 were also dominant values among Academic Emergency physicians in the United States (59%), only 1 percent of them had h-index higher than 24 and 5% had h-index higher than 13 (DeLuca et al., 2013). The median h-index of Anaesthesia researchers in the UK equaled 13 and the median g-index was 22 (Moppett & Hardman, 2011). Theh-index and g-index of faculty members at an Iranian university were reported so that the highest h and g indices (both 16) belonged to a nephrologist and the second place belonged to a microbiologist with h and g equaled 13 and 16, respectively. In this study h-index of 56% of faculty members was 0 (Gorgi et al., 2010). The top h-index was 17 for faculty members at Kerman University in Scopus and 46 percent had h-index of 0 (Okhovati et al., 2015).

Iranian Sports researchers with the most papers published in WOS were compared with other the most prolificSport researchers all around the world, the study revealed that the highest h-index for Iranian researchers was7 whereas the top h-index for Sport researchers in the WOS was 51. The difference between the mean of h-indecies for Iranian (4) and non-Iranian researchers (37.4) was significant (Yusefi, Zarki, & Sharifian, 2014).

According to multivariate regression, scientific age was statistically significant across h-index and g-index but not ar-index. A significant difference was observed among academic ranks. This difference was significant between instructors and assistant professors. Professors received higher scores than associate professors and assistant professors, across h-index and g-index significantly. However, ar-index across associate professors received higher scores than professors. Since ar-index can be decreased duringthe time, it implies that professors have less motivation than associate professors to publish and being cited.

Ranking Iranian biomedical research centers according to h, g, r, m and a-indices, Mahmudi, Tahamtan, Sedghi and Roudbari (2015) found the highest correlation between g-index and r-index in both WOS and Scopus. Furthermore, he highest overlapof the 10 top IBRCs was between g and h in WOS (100%) and between g and r (90%) and h and r (90%) in Scopus.

Adjusted regression demonstrated that among different academic ranks, professors had chance to significantly enhance their h-index. Rad et al. (2010) reported a significant relationship between h-index and academic rank. Multivariate logistic regression analysis demonstrated that h-index and number of publications were the best predictors of academic rank. Other studies confirmed the relationship between academic rank and h-index, Lopez, Susarla, Swanson, Calotta, and Lifchez (2015) put forward that h-index was strongly correlated with academic rank. According to Svider, et al. (2013) h-index values of academic otolaryngologists were higher in persons with increased academic rank among the levels of assistant professor, associate professor, and professor. The H-index of plastic surgeons was correlated with academic rank (Therattil et al., 2014). Lopezet al. (2015) also confirmed the strong correlation between h-index and academic rank when calculating the h-index for Full-Time Academic Hand Surgeons Affiliated With Fellowship Programs.

In summary, our study indicated that improving research performance measures is associated with academic ranking and scientific age of Iranian epidemiologists. However, h-index and g-index found to be more associated with academic ranks and scientific age than ar-index. Our findings confirmed earlier discussions on the role of academic ranking and scientific age of individual researchers with their scientific impact and reputation.
